# Domestic dogs (*Canis lupus familiaris*) are sensitive to the correlation between pitch and timbre in human speech

**DOI:** 10.1007/s10071-021-01567-4

**Published:** 2021-10-29

**Authors:** Sasha K. Sturdy, David R. R. Smith, David N. George

**Affiliations:** 1 Department of Psychology, University of Hull, Hull, HU6 7RX UK; 2 Present Address: Humberside Police, Hull, UK

**Keywords:** Dog, Speech, Glottal-pulse rate, Pitch, Vocal-tract length, Timbre

## Abstract

The perceived pitch of human voices is highly correlated with the fundamental frequency (*f*0) of the laryngeal source, which is determined largely by the length and mass of the vocal folds. The vocal folds are larger in adult males than in adult females, and men’s voices consequently have a lower pitch than women’s. The length of the supralaryngeal vocal tract (vocal-tract length; VTL) affects the resonant frequencies (formants) of speech which characterize the timbre of the voice. Men’s longer vocal tracts produce lower frequency, and less dispersed, formants than women’s shorter vocal tracts. Pitch and timbre combine to influence the perception of speaker characteristics such as size and age. Together, they can be used to categorize speaker sex with almost perfect accuracy. While it is known that domestic dogs can match a voice to a person of the same sex, there has been no investigation into whether dogs are sensitive to the correlation between pitch and timbre. We recorded a female voice giving three commands (‘Sit’, ‘Lay down’, ‘Come here’), and manipulated the recordings to lower the fundamental frequency (thus lowering pitch), increase simulated VTL (hence affecting timbre), or both (synthesized adult male voice). Dogs responded to the original adult female and synthesized adult male voices equivalently. Their tendency to obey the commands was, however, reduced when either pitch or timbre was manipulated alone. These results suggest that dogs are sensitive to both the pitch and timbre of human voices, and that they learn about the natural covariation of these perceptual attributes.

## Introduction

Pet dogs are most commonly kept as a source of companionship (Bennett and Rohlf 2007) and many owners anthropomorphize their dogs, considering them to be members of their family (Albert and Bulcroft 1987, 1988). The relationship between dogs and their owners is so close that about one-half of all dog owners share a bed or bedroom with their pets (Shepard 2002; Smith et al. 2017). Many owners believe that the love they have for their dogs is reciprocated (Serpell 1996, 2003; Wynne 2019) and see their pets as friends (Stallones et al. 1988) and confidants (Cassels et al. 2017; Evans-Wilday et al. 2018). Given the proximity in which dogs and people live, it is inevitable that dogs receive a great deal of exposure to human speech from shortly after birth. Much of dogs’ experience of human speech certainly results from incidental, or indirect, exposure to human-to-human communication and background noise from audio-visual devices in the home such as radio and television sets, but people also engage in a great deal of dog-directed speech (Mitchell and Edmonson 1999; Stallones et al. 1988). It is not surprising, therefore, that dogs have been found to be sensitive to various properties of human speech.

Dogs appear to recognize that speech is produced by humans and is distinct from dog vocalizations. Dogs and human infants show similar gazing patterns in preferential looking paradigms and display evidence of intermodal matching. When presented simultaneously with pictures of a dog and a human face along with either speech or a bark, both look towards the picture that matches the sound (Gergely et al. 2019). Dogs can discriminate between their owner and someone unknown to them based only on their voice (Gábor et al. 2019), and can differentiate between unfamiliar voices (Root-Gutteridge et al. 2019). They are able to match photographs of faces displaying emotional expressions (happy or playful vs. angry or aggressive) to appropriate vocalizations for both human and dog faces (Albuquerque et al. 2015). Dogs can discriminate between the lexical content of speech; some have been able to recognize in excess of 1000 words as referents for specific objects (Kaminski et al. 2004; Pilley and Reid 2011). As well as the lexical content, dogs are sensitive to ‘human’ qualities of voices; they respond poorly to commands delivered in dehumanized computer speech (Gibson et al. 2014). Intonation of commands affects the accuracy of dogs’ responses (Mills et al. 2005; Scheider et al. 2011), and dogs pay more attention to pet-directed speech, characterized by high pitch and exaggerated affect, than to adult-directed speech (Benjamin and Slocombe 2018, but see also Ben-Aderet et al. 2017; Jeannin et al. 2016). Different neural mechanisms of speech processing in dogs separately analyse and integrate lexical and intonational information (Andics et al. 2016) and hemispheric lateralization of the processing of phonemic and prosodic content of speech may be similar in dogs and humans (Ratcliffe and Reby 2014).

Humans can discriminate between male and female voices from a few months of age (Jusczyk et al. 1992; Miller et al. 1982) and dogs are also sensitive to speaker sex. Ratcliffe et al. (2014) presented dogs binaurally with either male or female speech while both a man and a woman were present in the same room. Although the dogs’ responses to this treatment were dependent upon the number of adult humans with whom the dogs lived, the sex of the speaker affected which person they spent more time looking at. Dogs that lived with more than two adults oriented towards the person whose sex matched the voice that they heard, whereas those who lived with a single adult looked towards the person whose sex was a mismatch to the voice. In either case, however, these biases suggest that the dogs spontaneously categorized the sex of the speaker.

Human speech sounds, and other mammalian vocalizations, are produced when air is pushed from the lungs and past the vocal folds in the larynx. The vocal folds vibrate open and closed, producing a series of discrete puffs of air known as glottal pulses. The rate at which the vocal folds vibrate—the glottal-pulse rate (GPR)—determines the fundamental frequency (*f*0) of the laryngeal sound source. The perceived pitch of voices is highly correlated with *f*0, which is in turn determined largely by the length and mass of the vocal folds. The vocal folds of males are typically about 60% larger than of those of females, resulting in an *f*0 that is about an octave lower (Titze 1989). Each time the vocal folds open and close, a glottal pulse moves into and through the space above the larynx, known as the supralaryngeal vocal tract. The length of the supralaryngeal vocal tract (vocal-tract length; VTL) affects the resonant frequencies (formants) of the voice, thus affecting the voice’s timbre. Increases in VTL result in lower, and less dispersed, formant frequencies; decreases in VTL result in higher, and more dispersed, formats frequencies (Fant 1970). VTL is highly correlated with speaker size (Fitch and Giedd 1999) and in men is approximately 20% longer than in women (Fant 1970). Not only do pitch and timbre both differ between male and female speakers, but they are also correlated with each other (Childers and Wu 1991). In humans, judgements of speaker sex are about equally influenced by these two perceptual attributes (Smith and Patterson 2005), and together they may be used to classify voices with near perfect accuracy (98.8%; Bachorowski and Orwen 1999).

Dogs can use the dispersion of formants in dog growls as cues for size. Taylor et al. (2011) manipulated recordings of growls to simulate formant dispersions associated with different VTLs. Using a preferential viewing paradigm, they showed that dogs matched these growls to appropriately sized taxidermy models of dogs. Dogs can also be trained to respond differentially to synthesized speech sounds with different *f*0 corresponding to male and female speech (Baru 1975). Although incidental learning has not been extensively investigated in dogs, there is evidence that they encode (Adachi et al. 2007; Kaminski et al. 2008) and learn about (Brogden 1939) covariation of stimuli even in the absence of reinforcement. The purpose of the experiment reported here was to determine whether dogs learn about the usual correlation between pitch and timbre in human voices as a result of their extensive exposure to speech.

**Fig. 1:**
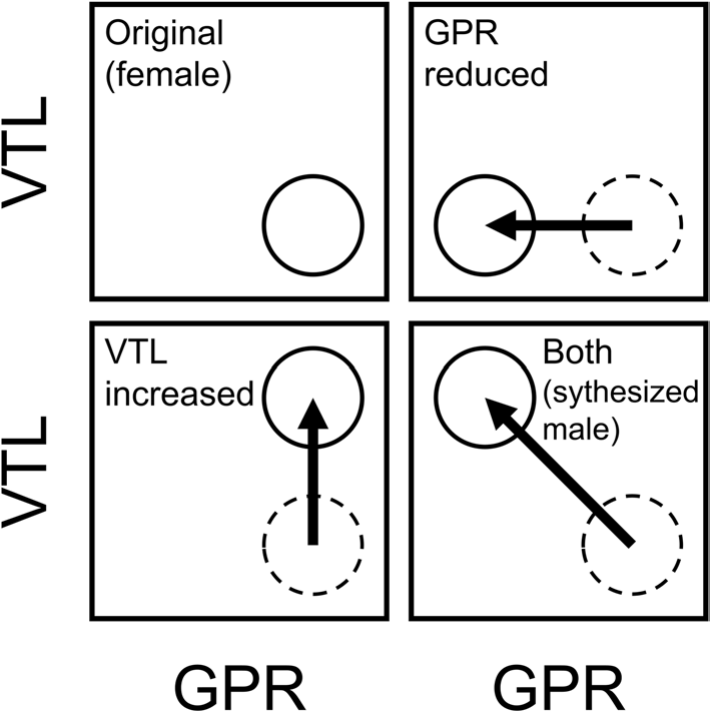
Schematic of the manipulations of the recordings. The original recordings of an adult female voice (top-left) was adjusted by either simulating a reduction in the glottal-pulse rate (GPR; top-right) alone, simulating an increase in the vocal-tract length (VTL; bottom-left) alone, or generating a synthesized adult male voice by performing both manipulations together (bottom-right). The dashed circle indicates the original position in GPR-VTL space of the adult female voice

We recorded an adult female voice issuing three commands (‘Come here’, ‘Lay down’, and ‘Sit’) and manipulated the recordings to simulate a reduction in GPR only (by reducing *f*0) leading to an adult male’s lower pitch for the command [mismatched to adult female timbre], simulate an increase in VTL only (by lowering the frequency and spacing of formants) leading to an adult male’s timbre for the command [mismatched to adult female pitch], or to simulate a male voice by both reducing the GPR and increasing VTL. Figure 1 shows a schematic of these manipulations. The unaltered and synthesized recordings were then played to ten dogs, and their responses were observed. Sensitivity to the correlation between pitch and timbre was expected to result in fewer correct responses to the commands when either *f*0 (simulating changes in GPR) or formant dispersion (simulating changes in VTL) was manipulated alone—which would result in a mismatch between the two perceptual attributes of pitch and timbre—compared to when either the voiced commands were unaltered (original adult female voice) or when they had both *f*0 and their formant dispersion changed (synthesized adult male voice).

## Methods

### Subjects

Ten pet dogs, all owned by members of the public, were recruited for the experiment (see Table 1 for details). The dogs were aged between 1 and 15 years (*M* = 6.7; SD = 5.2) and six were male. One of the dogs (Skye) was familiar with the experimenter. Prior to the start of the experiment, each dog’s owner confirmed that it had been trained with each of three commands (‘Come here’, ‘Lay/lie down’, and ‘Sit’), had good hearing, and was sufficiently mobile to respond to the commands. Before the first sessions of testing, the experimenter informally confirmed that the dog would respond appropriately to her voice by issuing the commands. All of the dogs were food motivated and were given occasional food rewards over the course of the experiment to help maintain their engagement with the task. In each case, the treats we used formed part of the dog’s normal diet and were supplied by the dog’s owner.

**Table 1:** Information about the dogs that participated in the experiment

Dog	Breed	Age (years)	Sex
Annie	Border Terrier	4	Female
Aukan	German Shepherd	9	Male
Balu	Lurcher	15	Male
Cooper	Labrador Retriever	7	Male
Hattie	Border Terrier	15	Female
Honey	Romanian Shepherd	3	Female
Iggy	Labrador Retriever	2	Male
Merckx	Belgian Malinois	1	Male
Puck	Belgian Malinois	2	Male
Skye	Border Collie	9	Female

### Materials

Audio recordings were made of a 21-year-old female’s voice (SKS) speaking the commands ‘Come here’, ‘Lay down’, and ‘Sit’ using an Audio-Technica AT2020 cardioid condenser microphone (Audio-Technica Ltd., Leeds, UK) connected to a PC running the Windows 7 operating system (Microsoft Corp., Redmond, WA) via a Yamaha AUDIOGRAM6 USB audio interface (Yamaha Corp., Hamamatsu, Japan). Audio signals were sampled at a frequency of 44.1 kHz in a 32-bit floating point format using the Audacity 2.2.2 recording and editing software (Audacity Team) and saved using the waveform audio file format (wav).

There are three ways of describing or viewing our manipulations. The anatomical processes or physical structures of GPR and VTL lead to acoustic variables such as *f*0 and formant dispersion. These are heard perceptually as pitch and timbre, respectively. To change the perceived pitch of a recorded speech sound, we may adjust its *f*0 which will simulate a particular GPR. If we wish to change a sound’s timbre, then we can adjust its degree of formant dispersion which simulates a particular VTL. It would only be possible to directly manipulate either GPR or VTL if we had a physical model of the speech production mechanism.

**Fig. 2:**
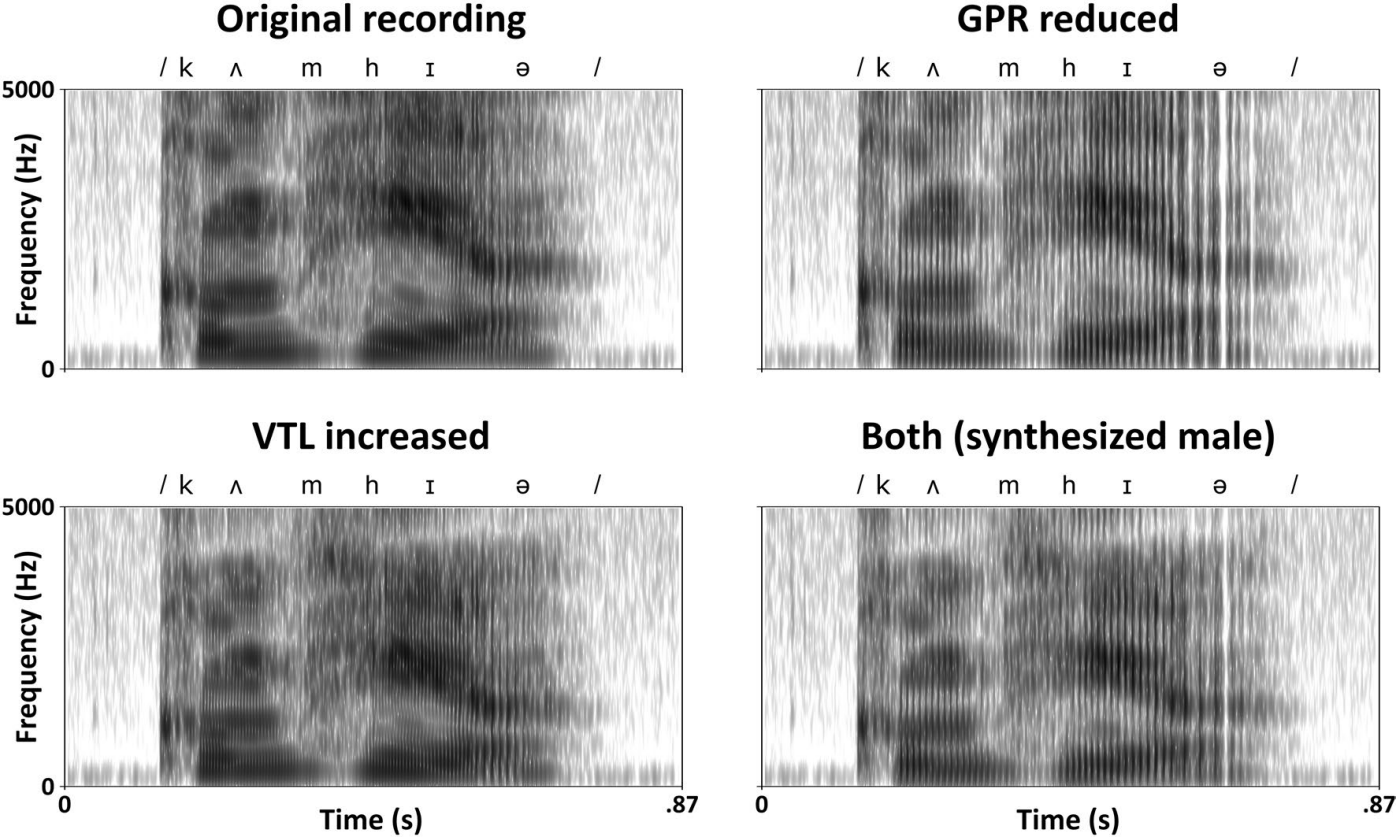
Spectrograms of recordings of the four versions of the ‘Come here’ command: the original recording (top-left), with simulated GPR reduced (top-right), with simulated VTL increased (bottom-left), and the synthesized male voice with both simulated GPR reduced and VTL increased (bottom-right). Darker greys correspond to higher energy values. The concentration of energy (darker greys) at certain frequencies marks the formants of speech—notice how they drop in frequency when the VTL is increased (within column change) denoting a change from a woman’s to a man’s VTL. The vertical striations mark the vocal fold vibrations—notice how the striations move apart when the GPR is reduced (within row change) denoting a change from a woman’s to a man’s GPR. The text above each spectrogram shows the approximate distribution of speech sounds over time transcribed using the International Phonetic Alphabet (International Phonetic Association 1999; ‘Come here’ → /kʌm/ /hɪə/)

The mean *f*0 of the spoken sections of ‘Come here’ was 208 Hz. A representative vowel /e/ in ‘Come h/e/re’ had formants of 447 Hz (*F*1), 2664 Hz (*F*2) and 3033 Hz (*F*3). The original sound files were manipulated to either decrease *f*0 by a factor of two (simulating a reduction in GPR) whilst leaving formant dispersion untouched, increase formant dispersion by 30% (simulating an increase in VTL) whilst leaving *f*0 untouched, or the manipulation was in both *f*0 and formant dispersion. These values were chosen to match the average difference in *f*0 and formant dispersion between adult male and female voices (Huber et al. 1999). The most natural sounding manipulated voiced commands had both *f*0 and their formant dispersion changed. Thus, the command ‘Come here’ converted to a male speaker by manipulating both simulated GPR and VTL had a mean *f*0 of 99 Hz and /e/ vowel formants of 348 Hz (*F*1), 2119 Hz (*F*2) and 2424 Hz (*F*3). Figure 2 shows spectrograms for the four versions of the ‘Come here’ command.

Similar adjustments (halving of *f*0; 30% increase in formant dispersion) were made to recordings of the commands ‘Lay down’ and ‘Sit’ to produce simulated reduced GPR only, simulated increased VTL only, and both simulated reduced GPR and increased VTL recordings. The mean *f*0 of the spoken sections of the original recording of ‘Lay down’ was 186 Hz. A representative long vowel /a/ in ‘L/a/y down’ had formants of 610 Hz (*F*1), 2174 Hz (*F*2) and 2743 Hz (*F*3). The mean *f*0 of the spoken sections of the original recording of ‘Sit down’ was 222 Hz. A presentative short vowel /I/ in ‘S/i/t’ had formants of 527 Hz (*F*1), 2169 Hz (*F*2) and 2828 Hz (*F*3). All values were calculated, and adjustments made, using the Praat programme (version 5.1.26; Boersma 2001). Sound recordings were played back using an iPhone X (Apple Inc., Cupertino, CA) connected to a Bluetooth speaker (Anker Soundcore, Anker Innovations Ltd., Hong Kong). Rewards, where given, were the standard training treats used by each dog’s owner.

Huber et al. (1999) reported that the mean fundamental frequency recorded from women (aged between 20 and 30 years with a mean age of 23.5 years) speaking at a comfortable effort level is 218 Hz with a standard deviation of 24 Hz (for the open backed unrounded vowel /ɑ/ sustained over 2–3 s). This is consistent with our adult female speaker’s mean fundamental frequency. *F*1 and *F*2 frequencies of the vowels (for instance /I/ in ‘Sit’) lie comfortably in the vowel ellipse shown in the classic Peterson and Barney (1952) study of vowels, and *F*1*–F*3 frequencies are all within the range of normal adult female speech reported by Kent and Vorperian (2018). The height of our speaker was 5′6″, which is near to the mean height for adult women born in the UK in 1996 (5′5″; NCD Risk Factor Collaboration 2016). Hence, we can assume that her VTL was of a typical length for an adult woman.

### Procedure

Each dog was tested in its own home and in the presence of its owner over 2 or 3 consecutive days. Testing lasted for approximately 30 min each day, not including occasion play breaks. Dogs tested over 2 days received 60 trials on each day, and those tested over 3 days received 40 trials on each day. On each trial, the experimenter stood approximately 1.5 m in front of, and facing, the dog with the Bluetooth speaker attached to a lanyard around her neck. The dog’s owner was positioned immediately behind the dog and held onto a lead attached to the dog’s collar. The owner ensured that the dog was standing at the start of each trial and then relaxed their grip on the lead to allow the dog to approach the experimenter (so that it might respond to ‘Come here’) when a command was played.

To orient the dog’s attention towards her at the beginning of each block of test trials, the experimenter gave the dog a treat. She then showed the dog a second treat in her left hand before closing her hand and raising it near to her mouth, obscuring her mouth from the dog’s view. On each trial, a recording of a command was played. If the dog’s response matched the command and was made within 5 s of the end of playback of the command, the trial was marked as correct. Otherwise, the trial was marked either as no response or, if the dog performed a response that did not match the command, as incorrect. To maintain the dog’s motivation to engage with the task, it was periodically rewarded with the treat held in the experimenter’s hand. A reward was given at the end of every fifth trial if the response on that trial was correct. If a correct response was not made on the fifth trial, then the next correct response was rewarded. In either case, the treat was then replaced, and counting was restarted. The longest run of trials between rewards for any dog was eight. At the end of each trial, the dog was returned to its standing starting position by its owner before the next trial commenced. This resulted in an interval of approximately 30 s from the start of one trial to the start of the next.

There were 12 types of trials generated by the combination of the three commands (‘Come here’, ‘Lay down’, and ‘Sit’) and the four different voices (original, simulated GPR reduced, simulated VTL increased, both simulated GPR reduced and VTL increased). Over the course of the experiment, each dog experienced each of the 12 trial types ten times, giving a total of 120 trials. The sequence in which trials were presented was randomized with the constraint that no command was given, or voice used, more than four times in succession within a session, and a combination of command and voice was not presented more than three times in succession. Before the beginning of the first day of testing, there were ten randomly selected practice trials to familiarize both the dog and its owner with the testing procedure. During these practice trials, no data were recorded.

### Data analysis

For each of the 12 combinations of command and voice condition, we collected data on ten trials. To assess the stability of performance across testing, data were partitioned into two blocks of 60 trials—five from each condition. These data were then used to calculate the proportion of trials on which each dog made the correct response in each condition across each block. Data were analysed using a four-way repeated measures analysis of variance (ANOVA) with the factors of Trial Block (first vs. second), Command (Come here; Lay down; Sit), GPR Condition (normal vs. reduced), and VTL Condition (normal vs. increased). If the dogs were sensitive to the normal correlation between GPR and VTL, we would expect performance to be worse when either GPR or VTL was manipulated alone when compared to the original (female) voice or to the synthesized male voice (where both GPR and VTL were altered). We therefore predicted an interaction between the factors of GPR condition and VTL condition. Dependent samples Student’s t tests were used to make pairwise comparisons between conditions where appropriate, and Šidák correction for multiple comparisons was applied. All analyses were conducted using IBM SPSS Statistics version 27 (IBM Corp., Armonk, NY).

## Results

**Fig. 3:**
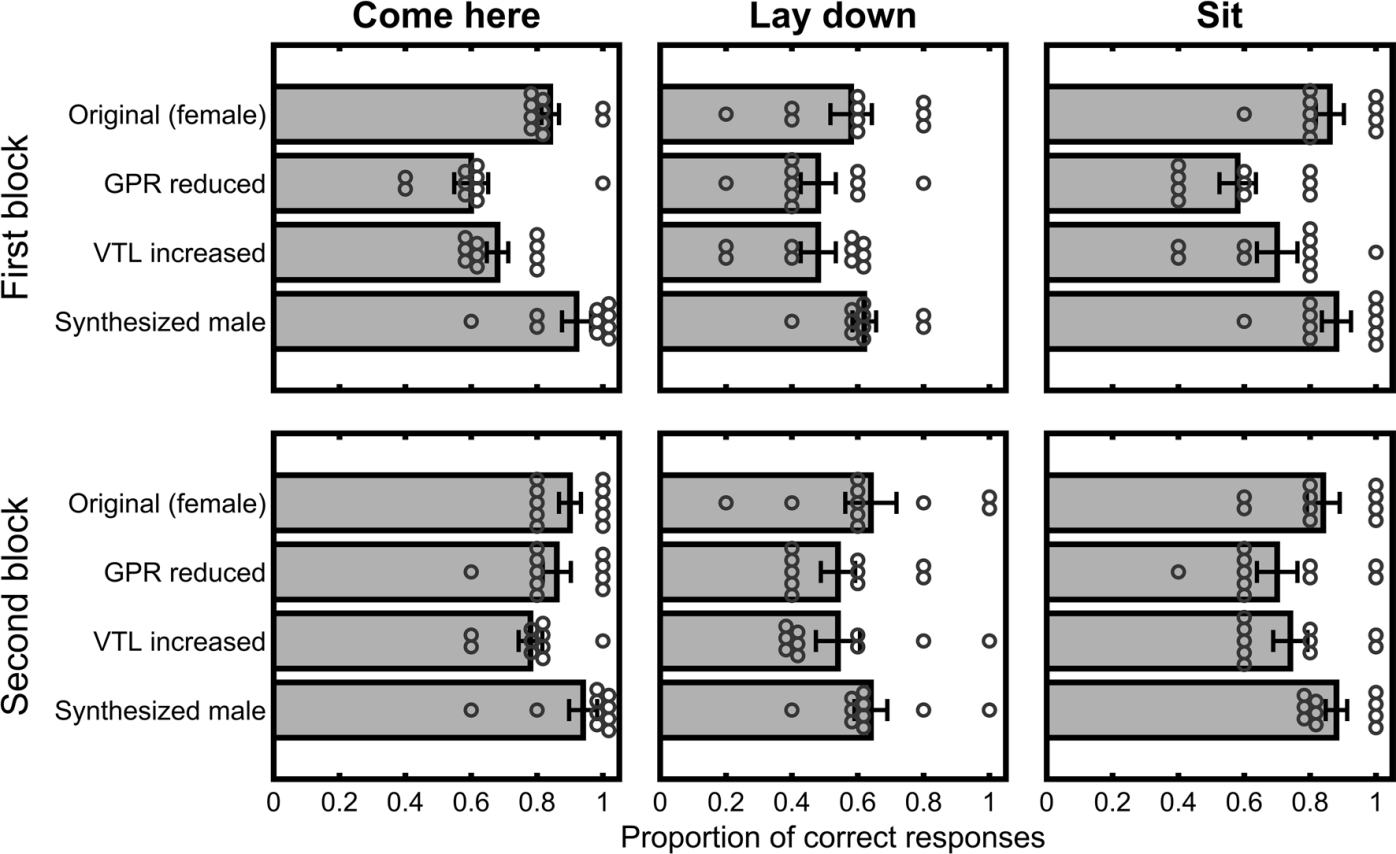
Mean proportion of correct responses that the dogs made to the commands. The three columns show the responses to each of the three commands (left: ‘Come here’; centre: ‘Lay down’; right: ‘Sit’). The top row shows data from the first block of 60 trials, and the bottom row shows data from the second block of 60 trials. Error bars indicate one standard error of the mean and the circles represent individual subjects’ data

Figure 3 shows the proportion of trial on which the dogs responded correctly in each of the voice conditions for the ‘Come here’ (left column), ‘Lay down’ (centre column), and ‘Sit’ (right column) commands. The top row of the figure shows performance across the first block of 60 trials, and the bottom row shows performance across the second block of trials. Dogs made fewer correct responses when either simulated GPR was reduced or simulated VTL was increased by themselves, relative to the original (female) voice. When both simulated GPR and VTL were adjusted together, to produce a synthesized male voice, the dogs’ responses were very similar to those that they made to the original voice. This pattern was evident for all three commands, but overall the dogs made fewer correct responses for the ‘Lay down’ command than either the ‘Come here’ or ‘Sit’ commands. Overall, the dogs made more correct responses on the second block of trials than on the first. Nevertheless, the effects of the voice manipulations, and the difference in performance across the three commands, was evident across both blocks of trials.

ANOVA confirmed these observations. There were main effects of Trial Block [*F*(1, 9) = 10.30; mean squared error, MSE = 0.025; *P* = 0.011; *η*_p_^2^ = 0.53; 90% confidence interval of the effect size (CI) (0.10, 0.71)], and Command [*F*(2, 18) = 66.01; MSE = 0.011; *P* < 0.0001; *η*_p_^2^ = 0.88; 90% CI (0.74, 0.91)], but no effect of either GPR Condition (*F* < 1), or VTL Condition [*F*(1, 9) = 4.46; MSE = 0.007; *P* = 0.064]. There was, however, a significant interaction of GRP Condition X VTL Condition [*F*(1, 9) = 161.83; MSE = 0.009; *P* < 0.0001, *η*_p_^2^ = 0.95, 90% CI (0.82, 0.97)]. Neither the Trial Block X GPR Condition X VTL Condition [*F*(1, 9) = 3.64; MSE = 0.029; *P* = 0.089], nor the Command X GPR Condition X VTL Condition [*F*(2, 18) = 3.33; MSE = 0.009; *P* = 0.059] interactions was significant. No other two-way interaction [largest *F*(1, 9) = 1.28; MSE = 0.029, *P* = 0.288], or three-way interaction (*F*s < 1) was significant, and neither was the four-way interaction (*F* < 1).

Post hoc pairwise t tests using Šidák correction for multiple comparisons (*α*_SID_ = 0.0085) revealed that performance for the original (female) voice (*M* = 0.78, SD = 0.035) was significantly better than for either the GPR reduced (*M* = 0.63, SD = 0.038) [*t*(9) = 9; *P* < 0.0001; mean difference = 0.15; 95% CI of mean diff. (0.11, 0.19)] or VTL increased (*M* = 0.65, SD = 0.048) [*t*(9) = 7.47; *P* < 0.0001; mean diff. = 0.12; 95% CI (0.09, 0.16)] voices. Performance for the synthesized male voice that had undergone both GPR reduction and VTL increase (*M* = 0.81, SD = 0.045) was also significantly better than for either the GPR alone [*t*(9) = 8.57; *P* < 0.0001; mean diff. = 0.19; 95% CI (0.14, 0.24)] or VTL alone [*t*(9) = 9.80; *P* < 0.0001; mean diff. = 0.16; 95% CI (0.12, 0.20)] adjusted voices. The GPR reduced voice did not differ statistically from the VTL increased voice [*t*(9) = 1.31; *P* = 0.22; mean diff. = 0.03], and the original voice did not reliably differ from the voice that had undergone both GPR reduction and VTL increase [*t*(9) = 2.18; *P* = 0.057; mean diff. = 0.04].

Dogs made fewer correct responses to the ‘Lay down’ command (*M* = 0.57, SD = 0.056) than to either ‘Come here’ (*M* = 0.82, SD = 0.029) [*t*(9) = 12.46; *P* < 0.0001; mean diff. = 0.25; 95% CI (0.20, 0.30); α_SID_ = 0.017], or ‘Sit’ (*M* = 0.77, SD = 0.055) [*t*(9) = 7.07; *P* < 0.0001; mean diff. = 0.21; 95% CI (0.14, 0.27)]. There was no reliable difference in the number of correct responses made to ‘Come here’ and ‘Sit’ [*t*(9) = 2.23; *P* = 0.052; mean diff. = 0.04].

## Discussion

We manipulated recordings of a female voice to generate a male-sounding voice by reducing *f*0 by about one-half (simulating a drop in GPR), thus lowering the perceived pitch, and by lowering the frequency and dispersion of formants to simulate an increase in VTL of approximately 30% (thus affecting the timbre of the voice). Dogs’ responses to commands issued by the original female voice and the synthesized male voice were very similar. When, however, dogs were played recordings for which either the simulated GPR or VTL had been adjusted alone, dogs produced fewer correct responses. This pattern of responding was reasonably stable across training. Dogs made more correct responses in the second block of 60 trials than they did in the first block, but there was no interaction between trial block and the manipulations to simulated GPR or VTL. The effect of trial block might be attributable to simple habituation to what must have been an unusual testing procedure for the dogs.

These results show that dogs are sensitive to the normal correlation between pitch and timbre in human voices. The decline in performance when these perceptual attributes were mismatched might have occurred because the dogs found it more difficult to extract the lexical content of the voice to identify the appropriate response. Alternatively, the unusual combination of pitch and timbre may have distracted them. That is, our results may be the result of a stimulus generalization decrement. Equivalent performance for the original female and synthesized male voices would indicate that such a generalization decrement was not due to the voices having *f*0 or formant dispersion outside the range of the dogs’ experience. Rather, it must have been based on the dogs’ knowledge that certain combinations of these features tend to co-occur.

While our dogs appeared to have learned the usual pairing between pitch and timbre that hold for men and women, our results do not allow us to identify the mechanism through which they did so. One possibility is that dogs learn the correlation directly as a consequence of both direct and indirect exposure to human voices from a variety of sources (e.g. in person, on TV and radio). Indeed, we have known for a long time that dogs can learn about the relationship between correlated neutral cues. In his original demonstration of sensory pre-conditioning, Brogden (1939) repeatedly presented dogs with the simultaneous sound of an electric doorbell and the flash of a light. In a second phase of the experiment, the bell alone was paired with a brief electric shock to the dogs’ left foreleg, conditioning a flexion response to the sound. At test, the light produced a similar flexion response despite never having been paired with the shock. In a control group of dogs that had not been given the initial simultaneous exposure to the two stimuli, the light provoked no response at test. This result is typically taken as evidence that the dogs learned an association between the bell and the light when they were presented together, and there is wider evidence that animals are sensitive to correlations between stimuli (e.g. Alloy and Tabachnik 1984; Santolin and Saffran 2018). In a similar way, dogs might learn that low- and high-pitched human voices are associated with different timbres. Alternatively, the learned relationship between pitch and timbre might be mediated through common associations with male and female categories. Internal representations of these categories might, in turn, be based upon learning about features that covary with sex in humans such as visual (including body-size), olfactory, and other auditory cues.

There is evidence that dogs organize people into male and female categories, and spontaneously categorize male and female voices (Ratcliffe et al. 2014). But it is not clear exactly which properties of the voices they use to do so. When Ratcliffe et al. presented dogs with a recording of a male or a female voice in the presence of a man and a woman, the dogs oriented towards the person whose sex matched the voice (or away from them, depending upon the number of adult humans that the dog lived with). The recordings that they used were taken from nine men and nine women, and on average the women’s voices had higher *f*0, and higher and more dispersed formant frequencies than the men’s voices. Although pitch and timbre can be used to predict speaker sex with near perfect accuracy (Bachorowski and Orwen 1999), other properties of voices differ between the sexes, such as articulation and intonation (Simpson 2009). Ratcliffe et al. describe no attempts to control for these factors. To assess the role of pitch and timbre in the spontaneous categorization of voices by dogs, one might adapt Ratcliffe et al.’s paradigm using stimuli manipulated in a similar manner to our own. We did not adopt that approach here for two reasons. First, the dogs’ responses to the voices in Ratcliffe et al.’s experiment were influenced by the number of human adults with which they lived and also the side to which the person of the matching sex was standing. Second, to determine whether *f*0 and formant dispersion affect categorization independently and/or jointly, a much more complex experimental design would have been required compared to the simple match/mismatch design used by Ratcliffe et al. Together, these issues meant that a very large sample of dogs would have been needed. Instead, the experiment reported here provides a first indication that dogs process the incidental properties of human voices such as pitch and timbre and may provide a foundation for future investigations of how they utilize this information, if indeed they do.

There are limitations to the current study. First, it is possible that our dogs’ performance was affected in some way by unintentional cues emitted by the experimenter. The voices that had undergone alterations to simulated GPR or simulated VTL alone did sound a little peculiar to our ears. It is therefore conceivable that the experimenter, when she heard these unusual voices, subconsciously reacted to them in some way that the dogs were able to detect, and which affected their responses to the commands. We took measures to limit this possibility; the experimenter adopted a fixed posture on each trial and obscured the lower part of her face with her hand. But it is impossible to be certain that we were entirely successful in eliminating all experimenter cues. Indeed, this is a problem for almost any hand-run experiment in which the experimenter cannot be fully blind to the conditions. Nevertheless, the stability of the interaction between simulated GPR and simulated VTL conditions across trials blocks in our experiment might provide some evidence that the effect was not due to an experimenter generated cue. If the experimenter did react to the unusual sounding voices, one would expect both that reaction, and the dogs’ reaction to it, to habituate over testing. That is, the effect should have become substantially smaller as testing went on. The absence of an interaction with trial block is, however, rather weak evidence by itself.

Second, while the recent trend in canine studies is towards larger sample sizes (see Arden et al. 2016), our sample of ten was quite small and we must consider whether our results can be generalized to pet dogs as a whole. Despite its size, our sample included members of seven breeds representing four of the eight breed groups recognized by the American Kennel Club (herding, hound, sporting, and terrier groups), across a wide range of ages (1–15 years) and included both males and females (6:4). Every one of the ten dogs made more correct responses to the original female and synthesized males voices than to the simulated GPR-alone and simulated VTL-alone manipulated voices. Further research with a much larger sample of dogs would be needed to assess whether breed, age, or sex influenced the size of our effect, but the consistency of our results is encouraging. Ratcliffe et al. (2014) have shown that the number, and sex, of the humans with which dogs live affects their responses to male and female human voices. It is quite possible that these factors would affect a dog’s experience of male and female voices and, hence, their knowledge of the relationship between pitch and timbre in human speech. Unfortunately, we did not record this information, but acknowledge that the influence of household composition might be another interesting topic for future research.

Finally, we recorded a single adult female voice and manipulated it to simulate the lower GPR and larger VTL associated with an adult male. The *f*0 and formant dispersion of our speaker’s voice were quite typical for an adult woman, and the synthesized male voice was produced by altering these variables according to the average difference between male and female speech. Nevertheless, we must also question the extent to which our results would generalize to other speakers, and especially to male voices that were manipulated to simulate the higher GPR and shorter VTL of female speakers. It might be particularly interesting to observe dogs’ responses to recording of natural voices which violate the normal coupling between pitch and timbre.

In conclusion, the experiment reported here provides the first evidence that domestic dogs learn about the relationship between pitch and timbre in human voices. These two properties of speech can be used to predict speaker sex with almost perfect accuracy, and dogs are known to be sensitive to speaker sex. It is, therefore, plausible that dogs’ categorization of speaker sex is based, at least partly, on the combination of pitch and timbre.

## Data Availability

The full dataset and stimuli are available on OSF: https://osf.io/msh2g/.
